# Koori Quit Pack: A Feasibility Study of a Multi-Component Mailout Smoking Cessation Support for Aboriginal and Torres Strait Islander People: “I Would Recommend it to Anybody. It’s Just so Much Easier.”

**DOI:** 10.1093/ntr/ntae106

**Published:** 2024-05-03

**Authors:** Michelle Kennedy, Raglan Maddox, Amanual Getnet Mersha, Catherine Chamberlain, Catherine Segan, Kerindy Clarke, Belinda Donaldson, Kayden Roberts-Barker, Joley Forster, Kade Booth, Billie Bonevski

**Affiliations:** College of Health Medicine and Wellbeing, University of Newcastle, Callaghan, NSW, Australia; Equity in Health and Wellbeing Research Program Hunter Medical Research Institute, University of Newcastle, New Lambton, NSW, Australia; National Centre for Epidemiology and Public Health, Australian National University, Canberra, ACT, Australia; College of Health Medicine and Wellbeing, University of Newcastle, Callaghan, NSW, Australia; Equity in Health and Wellbeing Research Program Hunter Medical Research Institute, University of Newcastle, New Lambton, NSW, Australia; Onemda Aboriginal and Torres Strait Islander Health and Wellbeing, Melbourne School of Population and Global Health, University of Melbourne, Melbourne, VIC, Australia; Judith Lumley Centre, School of Nursing and Midwifery, La Trobe University, Melbourne, VIC, Australia; Ngangk Yira Research Centre for Aboriginal Health and Social Equity, Murdoch University, Perth, WA, Australia; Cancer Council Victoria, Melbourne, VIC, Australia; Centre for Health Policy, Melbourne School of Population and Global Health, University of Melbourne, Melbourne, VIC, Australia; Medibank Private, Sydney, NSW, Australia; Victorian Aboriginal Community Controlled Health Organisation, Collingwood, VIC, Australia; College of Health Medicine and Wellbeing, University of Newcastle, Callaghan, NSW, Australia; College of Health Medicine and Wellbeing, University of Newcastle, Callaghan, NSW, Australia; College of Health Medicine and Wellbeing, University of Newcastle, Callaghan, NSW, Australia; Equity in Health and Wellbeing Research Program Hunter Medical Research Institute, University of Newcastle, New Lambton, NSW, Australia; Flinders Health and Medical Research Institute, College of Medicine and Public Health, Flinders University, Bedford Park, SA, Australia

## Abstract

**Introduction:**

Smoking is the leading cause of preventable death among Aboriginal and Torres Strait Islander people.

**Aims and Methods:**

The *Koori Quit Pack* study aimed to assess the feasibility of a multi-component mailout smoking cessation intervention to reduce smoking among Aboriginal and Torres Strait Islander people. A non-randomized, single-group feasibility study was conducted among Aboriginal and Torres Strait Islander people who reported current smoking. The intervention package included information pamphlets and resources on quitting, referral offer to Aboriginal Quitline and optional free Nicotine Replacement Therapies (NRT). Follow-up was conducted at 2-week, 6-week, 10-week, and 6-month post-recruitment. Feasibility outcomes were recruitment and retention rates, uptake of intervention components, and smoking abstinence at 6-week follow-up (primary endpoint). Cessation outcomes were analyzed using both a complete case analysis and intention-to-treat approach.

**Results:**

165 participants were recruited, 111 (67.3%), 79 (47.9%), 59 (35.8%), and 94 (57%) participants completed the 2-week, 6-week, 10-week, and 6-month follow-up. At 10-week follow-up, 40.7% of participants used pamphlets and booklets, 13.6% used Quitline and > 90% used NRT. At 6-week follow-up, 87.3% reported a quit attempt and 46.8% sustained quitting. 46.8% were continuously smoke-free at the 6-week timepoint. The complete case analysis and the intention-to-treat analysis at 6 months show a 7-day self-reported point prevalence abstinence of 34% and 19.4% respectively.

**Conclusions:**

The *Koori Quit Pack* mailout smoking cessation program was feasible to support Aboriginal and Torres Strait Islander people. The intervention resulted in a high smoking cessation rate and should be upscaled, implemented, and evaluated nationally.

**Implications:**

Aboriginal and Torres Strait Islander people are disproportionately impacted by tobacco-related harms; however, the majority want to quit or wish they never took up smoking. Mailout cessation support is feasible, overcomes access barriers to evidence-based support and increases quitting success. We recommend a national mailout smoking cessation program is implemented for, and by Aboriginal and Torres Strait Islander people to accelerate declines in smoking prevalence to eliminate tobacco-related death and disease.

## Introduction

Indigenous people, globally, continue to be impacted by commercial tobacco harms. This was embedded through colonization, such as the rationing of tobacco in lieu of wages,^1^ and direct targeting by the Tobacco Industry.^2^ In Australia, commercial tobacco continues to cause harm to Aboriginal and Torres Strait Islander people, communities, and culture as it is attributed to in excess of 10 000 deaths over the past decade.^3^ Smoking remains the leading cause of preventable death of Aboriginal and Torres Strait Islander people.^3^ While overall smoking prevalence has declined, 40.2% of Aboriginal and Torres Strait Islander adults smoke daily.^4,5^ Preventing tobacco initiation and providing cessation support are essential in reducing smoking prevalence and tobacco-related disease and death. Reducing smoking prevalence can improve population health, lead to significant economic savings,^6,7^ as well as uphold the right to health recognized by international human rights law.^,8^ Quitting tobacco offers immediate and long-term health advantages, contributing to healthier outcomes and helping to alleviate the burden of tobacco-related diseases.

The Australian Government’s National Tobacco Strategy 2023–2030 was recently launched, following the National Tobacco Strategy 2012–2018.^9^ The new Strategy explicitly includes Aboriginal and Torres Strait Islander people as a priority group, as well as people living in remote areas, pregnant people, young people and people in the penal system. This strategy complements a range of other strategies and plans including the National Aboriginal and Torres Strait Islander Health Plan 2013–2023,^10^ the National Closing the Gap Health Campaign, and the National Preventative Health Strategy 2021–2030.^11^ The National Preventative Health Strategy includes a reduction target of 27% prevalence among the Aboriginal and Torres Strait Islander population.^11^ While these new strategies and targets are promising, greater investment to accelerate the reduction in tobacco use among Aboriginal and Torres Strait Islander people is urgently required. However, there is limited evidence on effective smoking cessation programs for Indigenous people globally^12^ to inform policy and practice.

In line with the Australian Government’s strategies, plans, and targets and the World Health Organization Framework Convention on Tobacco Control (WHO FCTC) which highlights the importance of Indigenous people and community in driving the development, implementation, and evaluation of tobacco programs,^13^ the *Koori (a term used to refer to Aboriginal people from New South Wales and Victoria) Quit Pack* study was launched. The *Koori Quit Pack* study aims to build Indigenous-led evidence on smoking cessation care through the design, development, and implementation of community-led research. In this article, we report findings from the feasibility study of a mailout smoking cessation package and smoking cessation outcomes of participants.

## Materials and Methods

### Research Team

Relationality is foundational to Indigenous research practice and paradigm.^14^ We recognize that our own lived experience and standpoint are central to this research including the conceptualization, development, conduct, and interpretation of findings. Our team embodies Aboriginal and Torres Strait Islander lived experience (MK, CC, KC, JF, and KRB), Indigenous lived experience (RM), expertise in Indigenous tobacco research (MK, CC, RM, AGM, CS, and BB), Aboriginal health services (BD), qualitative research (MK, KB, and CC), and tobacco behavioral counseling (JF and CS). This study privileges Indigenous knowledge and recognizes the ongoing scientific rigor of Aboriginal and Torres Strait Islander people and their knowledge systems.

### Governance

Aboriginal and Torres Strait Islander people have led and informed all aspects of this study from design to dissemination. The *Koori Quit Pack* study arose from community priorities identified by the Aboriginal Health & Medical Research Council (AH&MRC) Tobacco Advisory Committee (TAC). The AH&MRC, Victorian Aboriginal Community Controlled Organization (VACCHO), New South Wales (NSW), Australian Capital Territory (ACT), and Victorian Quitline’s led the study design. Partnering organizations have overseen the data collection, analysis, interpretation, and reporting to ensure appropriate community involvement. Preliminary findings were shared with Aboriginal Community Controlled Health Services (ACCHS) across NSW and Victoria to assist ensure appropriate interpretation, framing, and contextualization of the findings.

### Ethics

Community-based approval and agreements were developed with partnering organizations to uphold data sovereignty principles.^15^ The project upholds ethical principles of research with Aboriginal and Torres Strait Islander peoples consistent with the National Health and Medical Research Council’s Guidelines for ethical conduct in Aboriginal and Torres Strait Islander health research, the AH&MRC ethical guidelines: Key principles (2020) V2.0. Ethical approvals were obtained from the AH&MRC Ethics Committee of NSW [1894/21] and the University of Newcastle [H-2022-0174]. All participants provided informed consent. The study is registered with the Australian New Zealand Clinical Trials Registry (#12622000654752). The reporting of the project has been conducted in line with the CONSIDER statement^16^ and ethical publishing practices.^17^

### Design and Setting

A non-randomized single-group, pre–post feasibility study was conducted to evaluate the feasibility of a mailout smoking cessation intervention for Aboriginal and Torres Strait Islander people in NSW, ACT, and Victoria, Australia. This study design was chosen as community co-design and identified that a control group would not be appropriate. Detailed study methods are provided in the published protocol.^18^ Baseline participant data and acceptability data are published in companion papers.^19,20^

### Participant Eligibility

Participants were eligible for the study if they met each of the following criteria: Are Aboriginal and/or Torres Strait Islander, self-reported current smoking (≥1 cigarette per day), were aged 16 years or older, resided in NSW, ACT, or Victoria, and intended to quit smoking within 30 days following study enrollment.

### Recruitment

Recruitment occurred between May and October 2022. Participants were recruited by self-referral using the following two strategies:

1. Online- paid advertisements on the social media platform (Facebook) and via posts shared by Aboriginal Community Controlled Health Organizations and Quitlines. Potential participants accessed detailed study information through a link in the advertisement.
2. Face-to-face- partnering ACCHSs provided potential participants with a recruitment card that included a QR code to the detailed study information sheet. Recruitment cards were disseminated by doctors and Aboriginal Health Worker and Practitioners during regular visits at the ACCHS, and at annual health checks.

For both recruitment strategies, potential participants provided consent and completed an online eligibility screen. Those eligible to participate provided their phone number and an Aboriginal research staff member (JF and KRB) then contacted the potential participant. Eligibility was confirmed over the phone, baseline data was elicited, and participants were provided access to the intervention.

### Intervention

#### Koori Quit Pack

**Figure 1.**
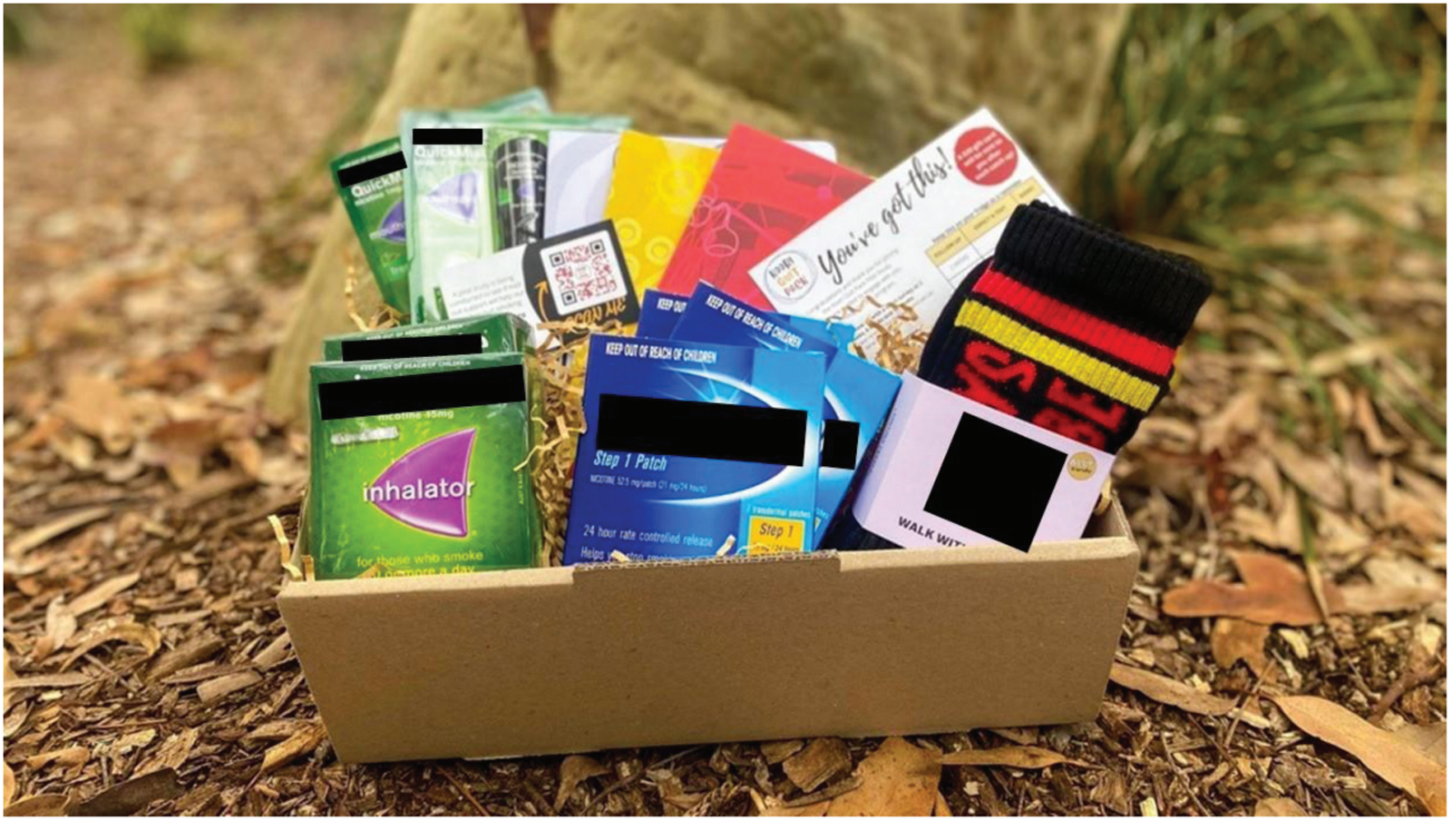
Image of the “Koori Quit Pack” mailout package contents.

Each participant received a mailout package that included pamphlets and resources on quitting, an information card on existing government-provided support options such as the MyQuitBuddy and the iCanQuit App (neither of which are specific to Aboriginal or Torres Strait Islander people), and Aboriginal Quitline webpage. *Koori Quit Packs* also included merchandise such as notebooks, socks, hats, and beanies at different time points to celebrate inclusion in the program and pride in culture (Figure 1). Participants were offered free Nicotine Replacement Therapy (NRT) and behavioral counseling as part of the intervention as detailed below, which was included in the mailout if accepted.

#### Offer of free NRT:

At baseline, participants completed a checklist to identify any NRT contraindications with assistance from Aboriginal research staff (JF and KRB). If no contraindications were present, participants were offered a 2-week supply of free combination NRT which was mailed to their residential address. Aboriginal research staff explained the different NRT formulations available, instructions for use, and how NRT would support quitting. The benefit of the information shared by the Aboriginal research staff is emphasized in the results of the study's acceptability publication.^20^ Participants selected NRT types based on their personal preference and previous experiences, strength of NRT was assessed using the Heaviness of Smoking Index at baseline. At 2-week follow-up, participants were offered an additional 4-week course of NRT and at 6-week follow-up, participants were offered an additional 4-week course to complete the 10-week NRT course recommended by the Royal Australian College of General Practitioners smoking cessation guideline.^21^ Participants could try different formulations of NRT during the intervention period.

#### Behavioral Counseling

At baseline and each follow-up, participants were offered a referral to Aboriginal Quitline services. The Aboriginal research staff explained the type of support provided by Aboriginal Quitline, and if the participant consented, an online referral was submitted directly to Quitline. Although not originally planned, due to the relationship built with participants and based on their requests, Aboriginal research staff also provided direct smoking cessation support to participants during recruitment and follow-up data collection points. Participants could also reach out to Aboriginal research staff via text to have any questions answered or request a phone call for behavioral counseling.

### Data Collection

Data collection was conducted by two Aboriginal research staff (KRB, Wiradjuri man and JF, Worimi woman) at baseline, 2, 6, and 10-week and 6-month follow-up. Data were collected via telephone interview and entered manually into the secure Research Electronic Data Capture (REDCap) system. Prior to the phone interview participants were sent a text message to advise the research team of an appropriate day and time for the phone call. Participants were sent up to three text messages to schedule data collection before being identified as lost to follow-up. Participants were provided with an AUD$30 gift card at each time point as reimbursement for their time. Participants who completed the 6-month follow-up were placed in a draw for an Apple iPad.

## Measures

Details of the development of the data collection tools, content, and measures are presented in the study protocol.^18^

### Sociodemographic and Mental Health and Well-Being Characteristics

At baseline, participants reported their age, gender, postcode, and highest level of education. Postcode was used to determine remoteness and relative socioeconomic disadvantage. Based on the 2016 Australian Bureau of Statistics socioeconomic index,^22^ a score of < 1000 indicated socioeconomic disadvantage, and a score of > 1000 indicated no socioeconomic disadvantage. Depressive symptoms were assessed using the nine-item Patient Health Questionnaire (PHQ-9) adapted for Aboriginal and Torres Strait Islander peoples.^23^ A cut-point score of 10 was used to determine the presence of symptoms of depression.

### Feasibility

Feasibility outcomes were determined through the assessment of participant recruitment rates, retention rates, and uptake of each component of the *Koori Quit Pack*. The number of participants who completed screening and baseline data collection was recorded. Intervention uptake was assessed by participant self-report of each component of the mailed *Koori Quit Pack* (MyQuitBuddy App, Quitline, Aboriginal Quitline webpages, Pamphlets or booklets, iCanQuit App, and NRT) at each follow-up point. Participants also self-reported on NRT the frequency and duration of use, safety and efficacy beliefs, and type preference. Adherence to NRT was determined based on duration and frequency of use at 6- and 10-week follow-up, with adherence defined as utilization of NRT at least five times per week for at least four weeks.^24^

### Smoking-Related Outcomes

Smoking-related outcomes assessed included number of quit attempts, self-reported continuous abstinence, self-reported 7-day point prevalence abstinence, changes in motivation, and confidence to quit smoking from baseline to the end of the intervention. Participants were asked to report quit attempts and continuous smoking cessation at each follow-up time point. At 6 months follow-up, participants were asked if they had smoked, even a puff, in the last 7 days to assess 7-day point prevalence abstinence, an item recommended by the Society for Research in Nicotine and Tobacco.^25^ Motivation and confidence to quit smoking were each assessed using a five-point Likert scale (not at all, slightly, moderately, very, and extremely motivated and confident). Ratings of motivation and confidence to quit were further categorized as low (not at all, slightly, moderately motivated and confident) or high (very and extremely motivated and confident).

### Data Analysis

Descriptive statistics were used to present the characteristics of participants enrolled in the study, participant recruitment rates, retention rate, and uptake of each component of the *Koori Quit Pack* intervention. Smoking cessation outcomes were analyzed in two ways. First, a complete case (per protocol^18^) approach was used where analysis was based on those actually participating in the study. Second, an intention to treat analysis was conducted whereby participants lost to follow-up were classified as continuing to smoke. Cross-tabulation with Pearson’s chi-square test was used to evaluate smoking cessation based on group differences such as depressive symptoms, level of nicotine addiction, adherence to NRT, combination NRT use, type of support used, socioeconomic disadvantage, remoteness, strength, and frequency of urges to smoke, level of motivation and confidence to quit smoking. Additional analysis was conducted to evaluate the association of e-cigarette use with combination NRT use, adherence, quit attempt, and quitting. Given the exploratory nature of this study, no adjustments for multiple comparisons were performed to evaluate the factors associated with smoking cessation. Analysis was performed using SPSS V27.0.

## Results

### Participant Characteristics

A total of 165 participants with mean age of 41.13 years (SD = 10.69) and 79.4% female completed baseline data collection. The majority resided in NSW (83%), and 57% resided in areas with greater sociodemographic disadvantage. Almost an equal number of participants resided in metropolitan areas and remote or rural areas of Australia. Symptoms of depression were reported by 18.4%, 15.5%, 16.7%, and 18.6% at baseline, 2-, 6-, and 10-week follow-up, respectively. At baseline, 10.3% of participants reported e-cigarette use, this number remained relatively consistent at the end of the study period (8.5%). Details of the sociodemographic characteristics of participants are presented in the baseline cohort profile study for the Koori quit pack.^19^

### Feasibility and Acceptability Outcomes

#### Participant Recruitment and Retention

**Figure 2.**
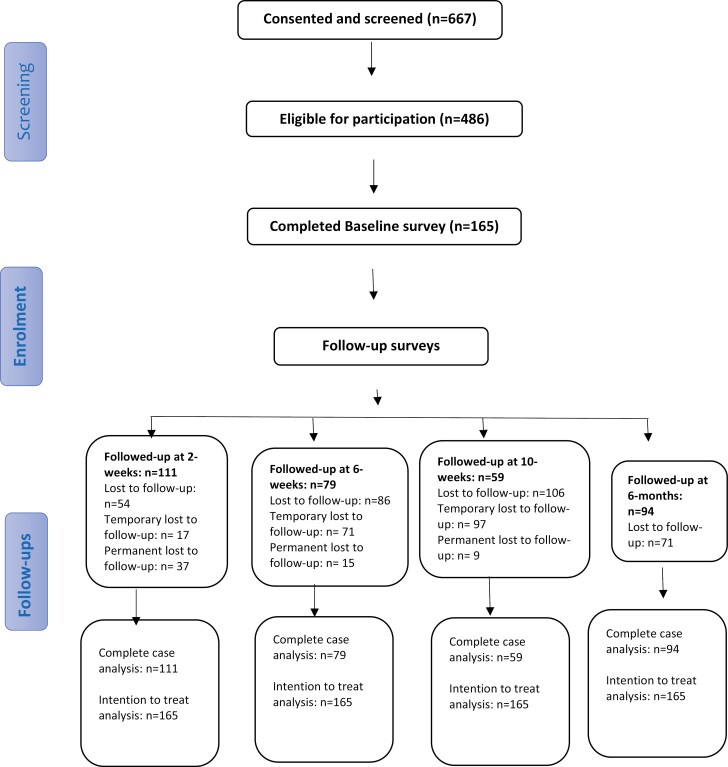
Flow diagram for the study participants

We aimed to recruit 100 participants.^26^ A total of 667 individuals consented to participate and underwent eligibility screening. Of these participants, 486 met the eligibility criteria and were asked to provide contact details for baseline data collection. Contact details for 234 participants were provided. Baseline data collection was completed by 165 (70.5%). Respectively, 111 (67.3%), 79 (47.9%), 59 (35.8%), and 94 (57%) of participants completed 2-week, 6-week, 10-week, and 6-month follow-up surveys (See Figure 2 which illustrates the study participant flow).

#### Acceptance and Use of the Koori Quit Pack Intervention Components

Table 1 details participant use of each component of the *Koori Quit Pack* intervention. Overall, during the intervention period, more than 90% of participants accepted and utilized NRT. At baseline, most participants requested and were sent nicotine patches (85.5%) and inhalers (69.7%). Nasal spray (28.5%), lozenges (27.3%), and gum (13.3%) were also frequently requested. Patches and inhalers remained the most preferred NRT products throughout the study and were used by 30.5% and 22.1% of participants at the 10-week follow-up. Of those who used NRT, 70% used combination NRT. Of the participants who completed the 10-week follow-up, 69.5% reported using NRT consistently throughout the 2-, 6-, and 10-week follow-up periods. Additionally, 33.9% of participants reported using a combination of NRT during the same periods. At 6- and 10-week follow-ups, 42.1% and 54.2% of participants respectively reported NRT adherence. No significant differences were identified for combination NRT adherence among participants who reported using e-cigarettes, and those who did not use e-cigarettes.

**Table 1.** Koori Quit Pack Component Utilization at 2-, 6-, and 10 Weeks

Components		2 weeks *N*(%)	6 weeks *N*(%)	10 weeks *N*(%)
NRT	Used any NRT			
	Yes	106(96.4)	76(97.4)	49(90.7)
	No	4(3.6)	2(2.6)	5(8.5)
	Type of NRT used			
	NRT: Nicotine patch	90(68.7)	59(55)	40(30.5)
	NRT: Nicotine gum	17(13)	17(13)	15(11.5)
	NRT: Lozenge	28(21.4)	22(16.8)	12(9.2)
	NRT: Mouth spray	29(22.1)	24(18.3)	15(11.5)
	NRT: Inhalers	69(52.7)	45(34.4)	29(22.1)
	Combination NRT use			
	Used Combination NRT	74(69.8)	53(69.7)	34(69.4)
	Used single NRT type	32(30.2)	23(30.3)	15(30.6)
	Frequency of NRT use			
	Used occasionally, 1–2 times a week	12(11.3)	7(9.2)	2(4.2)
	Used 3–4 times a week but not all doses	16(15.1)	10(13.2)	4(8.3)
	Occasionally missed a dose	12(11.3)	9(11.8)	11(22.9)
	Used most doses, every day	66(62.3)	50(65.8)	31(64.6)
	Duration of NRT use			
	For less than 1 week	**—**	5(6.6)	0(0)
	For 1–2 weeks	**—**	13(17.1)	5(10.2)
	For 2–4 weeks	**—**	17(22.4)	4(8.2)
	For 4–6 weeks	**—**	26(34.2)	11(22.4)
	Longer than 6 weeks	**—**	15(19.7)	29(59.2)
	Adherence to NRT			
	Yes	**—**	32(42.1)	26(54.2)
	No	**—**	44(57.9)	22(45.8)
Behavioral supports	Accepted Quitline referral			
	Yes	18(16.4)	13(16.5)	8(13.6)
	No	92(83.6)	66(83.5)	51(86.4)
	Used Quitline (having at least one call)			
	Yes	10(9)	9(11.4)	8(13.6)
	No	101(91)	70(88.6)	51(86.4)
	Used aboriginal Quitline webpages			
	Yes	17(15.3)	14(17.7)	8(13.6)
	No	94(84.7)	65(82.3)	51(86.4)
	Used pamphlets and booklets			
	Yes	65(58.6)	39(49.4)	24(40.7)
	No	46(41.4)	40(50.6)	35(59.3)
	Used my Quitbuddy app			
	Yes	34(30.6)	21(26.6)	9(15.3)
	No	77(69.4)	58(73.4)	50(84.7)
	Used iCanQuit s			
	Yes	2(1.8)	1(1.3)	2(3.4)
	No	109(98.2)	78(98.7)	57(96.6)

Pamphlets and booklets were used by 58.6% and 40.7% of participants at 2- and 10 weeks of the follow-up, respectively. Acceptance of referrals and usage of Quitline was utilized by 13.6% by the 10-week follow-up. Aboriginal Quitline webpages were accessed by 15.3% of participants at 2 weeks and 13.6% at 10-week follow-up. The My QuitBuddy mobile app was accepted and used by 30.6% of the participants at 2-week follow-up, and 15.3% by the 10-week follow-up. The iCanQuit mobile app was used by two participants (1.7%). (Table 1) One behavioral and pharmacological support was utilized by 72.6% and 59.2% at the 2-week and 10-week follow-ups, respectively. At the 2- and 10-week follow-up, respectively, 27.4% and 40.8% of participants used NRT without the other resources.

### Attitudes, Beliefs, and Motivation to Quit

Participant attitudes, beliefs, and motivation to quit are provided in Table 2. Throughout the study period, almost all participants believed smoking cessation would have a positive impact on their health. At baseline, the participants showed high levels of confidence and motivation to quit smoking, with a mean motivation score of 8.03(SD = 1.66) out of 10. Their level of motivation and confidence in quitting smoking remained high during the follow-up periods and over a third (37.3%) of the participants made a quit plan. Participants who made a quit plan found it to be helpful. At baseline, most participants strongly agreed or agreed that NRT was safe (80.6%) and effective (70.3%), and this increased to 93.2% and 89.8% at the end of the intervention period (Table 2).

**Table 2.** Attitudes, Belief and Motivation to Quit at Baseline, 2-, 6-, and 10 Weeks

	Baseline	2 weeks	6 weeks	10 weeks
	*N*(%)	*N*(%)	*N*(%)	*N*(%)
Quitting improves my health				
Strongly agree	136(82.4)	98(88.3)	73(92.4)	55(93.2)
Agree	28(17)	12(10.8)	6(7.6)	4(6.8)
Neutral	1(0.6)	0(0)	0(0)	0(0)
Strongly disagree	0(0)	1(0.9)	0(0)	0(0)
Motivation to quit				
Not at all motivated	—	1(0.9)	2(2.5)	1(1.7)
Slightly motivated	—	4(3.6)	0(0)	2(3.4)
Moderately motivated	—	15(13.5)	13(16.5)	5(8.5)
Very motivated	—	39(35.1)	30(38)	19(32.2)
Extremely motivated	—	51(45.9)	34(43)	32(54.2)
Refused to answer	—	1(0.9)	0(0)	0(0)
Confidence in quitting smoking				
Not at all confident	4(2.4)	3(2.7)	1(1.3)	1(1.7)
Slightly confident	13(7.9)	4(3.6)	3(3.8)	4(6.8)
Moderately confident	67(40.6)	25(22.5)	11(13.9)	6(10.2)
Very confident	55(33.3)	38(34.2)	31(39.2)	12(20.3)
Extremely confident	22(13.3)	40(36)	33(41.8)	36(61)
Don’t know	4(2.4)	1(0.9)	0(0)	0(0)
Made a quit plan				
Yes	—	30(27)	21(26.6)	22(37.3)
No	—	81(73)	58(73.4)	37(62.7)
Quit plan was helpful				
Yes	—	26(86.7)	20(95.2)	20(90.9)
No	—	4(13.3)	1(4.8)	2(9.1)
NRT is safe				
Strongly agree	54(32.7)	59(53.2)	53(67.1)	43(72.9)
Agree	79(47.9)	36(32.4)	20(25.3)	12(20.3)
Neutral	32(19.4)	16(14.4)	6(7.6)	4(6.8)
NRT is effective				
Strongly agree	46(27.9)	59(53.2)	50(63.3)	40(67.8)
Agree	70(42.4)	44(39.6)	25(31.6)	13(22)
Neutral	49(29.7)	7(6.3)	4(5.1)	6(10.2)
Strongly disagree	0(0)	1(0.9)	0(0)	0(0)

### Smoking Cessation Outcomes

Table 3 provides smoking cessation outcomes at each follow-up point for both the complete case and intention to treat analyses.

**Table 3.** Smoking Cessation Outcomes at Each Follow-up Point

Outcomes		Complete case analysis				Intention to treat analysis (*n* = 165)			
		2 weeks (*n* = 111)	6 weeks (*n* = 79)	10 weeks (*n* = 59)	6 months (*n* = 94)	2 weeks (67.3%)	6 weeks (47.9%)	10 weeks (35.8%)	6 months (57%)
Quit attempt	Yes	89(80.2%)	69(87.3%)	45(76.3%)	83(88.3%)	89(53.9%)	69(41.8%)	45(27.3%)	83(50.3%)
	No	16(14.4%)	9(11.4%)	6(10.2%)	11(11.7%)	16(9.7%)	9(5.5%)	6(3.6%)	11(6.7%)
	Others^*^	6(5.4%)	1(1.3%)	8(13.6%)	0(0%)	6(3.6%)	1(0.6%)	8(4.8%)	0(0%)
Continuous abstinence	Yes	46(41.4%)	37(46.8%)	33(55.9%)	20(21.3%)	46(27.9%)	37(22.4%)	33(20%)	20(12.1%)
	No	65(58.6%)	42(53.2%)	26(44.1%)	74(78.7%)	65(39.4%)	42(25.5%)	26(15.8%)	74(44.8%)
7-day PPA	Yes	**—**	**—**	**—**	32(34%)	**—**	**—**	**—**	32(19.4%)
	No	**—**	**—**	**—**	62(66%)	**—**	**—**	**—**	62(37.6%)

^*^Cut down the number of cigarettes smoked per day.

#### Complete Case Analysis

At 2 weeks follow-up, 80.2% of participants made a quit attempt and 41.1% had remained smoke-free for the entire 2 weeks. At the 6-week follow-up, 87.3% of the participants reported a quit attempt and 46.8% remained smoke-free since their last follow-up. At the 10-week follow-up, 76.3% reported a quit attempt, and 55.9% remained smoke-free since their last follow-up. At the end of the follow-up period (6 months), 88.3% of participants reported making a quit attempt since their last follow-up and 21.3% remained continuously smoke-free for the entire 6 months of follow-up. The 7-day point prevalence of abstinence (PPA) was found to be 34% at the 6-month follow-up (Table 3). The chi-squared test indicated a significant association between the level of motivation and depression symptoms with 7-day PPA at 6 months. The vast majority of participants who were found to be smoke-free at 6 months had no depression symptoms 30(93.8%; Chi-squared *p*-value = .021). Similarly, participants who were found to be smoke-free at 6 months had a high level of motivation to quit smoking 32(100%) (Chi-squared *p*-value = .048; Table S1). Participants who did not use e-cigarettes to quit smoking reported 47.8% continuous abstinence outcome, and those who did use e-cigarettes reported 41.7% at 6 weeks follow-up. At the 10- week follow-up the continuous abstinence rate was 59.3% and 20%, respectively, among those who did not use e-cigarettes to quit smoking and those who did use e-cigarettes.

#### Intention to Treat Analysis

Intention to treat analysis considered dropouts as continuing to smoke. Based on this the rate of self-reported continuous abstinence was found to be 27.9% (2-week), 22.4% (6-week), 20% (10-week), and 12.1% (6-month) at follow-up. The 7-day self-reported PPA was 19.4% at 6-month follow-up.

## Discussion

The *Koori Quit Pack* study is among the first Indigenous-led and governed studies to explore mailout smoking cessation support with optional NRT. The findings from this study indicate that multi-component mailout smoking cessation support for Aboriginal and Torres Strait Islander people is feasible and can enhance the accessibility of smoking support. While mailout NRT^27^ and printed self-help materials^28^ have been shown to be effective in other populations, this study provides evidence that phone support from Aboriginal staff coupled with the provision of mailout cessation supports was highly feasible and acceptable, demonstrated by high recruitment rates, uptake of NRT and cessation outcomes.

Participant recruitment to the study occurred at a much faster rate than anticipated,^18^ demonstrating the acceptability of the comprehensive community-led recruitment processes. While we did not track the relative effectiveness of the variety of recruitment approaches, the recruitment approach was designed by Aboriginal and Torres Strait Islander people and communities and informed by evidence from other trials.^29,30^ Recruitment to the study was higher in NSW than Victoria and the ACT, which may reflect variation in awareness and engagement with the study within different Aboriginal communities and ACCHOs, and also the proportionally higher population of Aboriginal and Torres Strait Islander people in NSW comparative to Victoria and the ACT.^31^ However, given the study ceased recruitment early as funding has been exhausted, participant recruitment from other states may have increased over time. While loss to follow-up ranged from 33%–64% of the baseline sample across the follow-up timepoints, this is in line with previous research^32^ and could be due to the online mode of recruitment and phone call follow-up.

The uptake of NRT was higher than expected given previous evidence reported only 37% of Aboriginal and Torres Strait Islander smokers had ever used NRT.^33^ Nicotine patches and inhalers were the most preferred formulations throughout the study, and just over half (54.2%) of participants self-reported adherence to NRT at 10 weeks follow-up. Participants reported positive attitudes toward the safety and effectiveness of NRT, with the vast majority using the product throughout the study. This finding is new and compliments previous evidence on the acceptability of NRT among Aboriginal and Torres Strait Islander people to support quitting.^33^ Further, it provides additional evidence of the need to address the barrier of cost of NRT to enhance cessation outcomes.^33^ In Australia currently, oral NRT is not subsidized under the pharmaceutical benefits scheme and patches are only subsided as a “sole” support.^34^ These limitations make accessing recommended cessation support, particularly combination NRT, costly. Acknowledging government strategies and targets to reduce smoking prevalence, there is an urgent need to ensure that Aboriginal and Torres Strait Islander people have access to evidence-based care, either through subsidized smoking cessation medications including all oral NRT, and/or block funding for community-led programs.^35,36^

The multi-component mailout intervention resulted in quit attempts and quit success among participants. Using the conservative intention to treat analysis, 50% of the sample had made a quit attempt at 6 months follow-up and 19.4% were smoke-free. Using the complete case analysis, 88.3% of participants had made a quit attempt at 6 months follow-up and 34% remained smoke-free. These findings align with evidence from mailout interventions in other countries and in non-Indigenous settings. A similar study conducted in Canada resulted in a more than two-fold 30-day smoking abstinence at 6 months among participants randomized to 5-week course of mailout nicotine patches as compared to no treatment control group.^27^ Another study conducted among patients waiting for elective surgery at Frankston Hospital in Australia showed an improved rate of smoking cessation among participants who received a mailout smoking cessation support with NRT and Quitline referral as compared to participants in the usual care arm.^37^ Our findings indicated higher successful quit rates than other smoking cessation studies among Aboriginal and Torres Strait Islander people, which have reported quit rates of 4.5%^38^ to 11%.^21^ A small number of participants reported using e-cigarettes during the study with limited cessation (10.3% baseline and 8.5% 6-month), which is higher than previously reported evidence.^39^ Acknowledging the higher use of e-cigarettes, it is important that future population health initiatives and cessation programs incorporate e-cigarette cessation as well.

The majority of participants in the *Koori Quit Pack* study who were successfully smoke-free at six months were both highly motivated and did not report depressive symptoms at baseline or throughout the study. This is in line with recent evidence among Aboriginal and Torres Strait Islander people who reported the negative effect of depression on quit attempts and sustained quitting.^40^ As well as international evidence on the correlation between depressive symptoms and continued smoking,^41^ this finding signifies the importance of addressing mental health and providing additional support for participants experiencing symptoms of depression during smoking cessation care. There is a need for a more holistic approach to smoking cessation care that integrates mental health services and support to identify and provide tailored support for individuals experiencing depression symptoms.^42,43^

## Strengths and Limitations

This study is the first study to evaluate the feasibility of mailout smoking cessation support for Aboriginal and Torres Strait Islander peoples, and as such, expands current knowledge of appropriate and meaningful smoking cessation supports. Findings highlight the importance of developing culturally responsive and Indigenous-led implementation studies that consider the unique challenges faced by Aboriginal and Torres Strait Islander peoples in their journey towards quitting smoking. Compared to previous similar trials conducted among Aboriginal and Torres Strait Islander peoples who smoke,^38,44^ this study successfully recruited a substantial number of participants from both metropolitan and remote or rural areas, and a high proportion of women. The high recruitment of women contrasts with other studies with Aboriginal and Torres Strait Islander people,^21,45^ however this could be influenced by the online mode of recruitment, as reported in other studies.^46^ While recruitment occurred quickly, there was a high dropout rate for baseline data completion. While it is unclear why this occurred, this could be due to the online mode of recruitment and participants not having the time or interest to complete a survey tool. The relationship and support offered by the Aboriginal research staff were considered essential in the acceptability of the intervention. Further details on this meaningful and imperative process can be found elsewhere.^20^ The main limitation of this study is that as a single-group trial, it is not powered to identify the various factors that may influence successful quitting, or the influence of the recruitment process from the intervention components. Nonetheless, our qualitative data suggests that participants found both recruitment and intervention to be acceptable and effective.^20^

Quit rates were not verified as the Aboriginal community partners indicated during project design that verification was not appropriate or feasible outside of a clinic setting. A mailout study conducted at a similar time also experienced low feasibility of biochemical verification.^47^ However, it does provide valuable insights into the feasibility and potential challenges of implementing smoking cessation interventions tailored to Indigenous communities. While a limitation of the study is the retention rate, which impacted the observed smoking cessation rates in the intention-to-treat analysis, the feasibility and acceptability of the method of recruitment and follow-up is promising for other interventions to support Aboriginal and Torres Strait Islander health. A future well-powered study is recommended to identify factors that optimize smoking cessation strategies and improve outcomes within Aboriginal and Torres Strait Islander peoples.

## Conclusion

Aboriginal and Torres Strait Islander people across age groups and geographical locations are seeking support and are highly motivated to quit smoking. NRT is widely acceptable and of interest to Aboriginal and Torres Strait Islander people but current provisions of NRT present barriers in access and affordability. Our study reported high quit rates through the provision of mailout combination of NRT and behavioral support by an Aboriginal research team. We recommend a national mailout smoking cessation program is developed and implemented for and by Aboriginal and Torres Strait Islander people to drive progress toward Government-developed reduced prevalence targets.

## Supplementary material

Supplementary material is available at *Nicotine and Tobacco Research* online.

ntae106_suppl_Supplementary_Material

## Data Availability

Relevant materials and data supporting the findings are included in the manuscript.

## Patient and Public Involvement

The data collection, analysis and reporting of this study is overseen by the Aboriginal Health & Medical Research Council Tobacco Advisory Committee to ensure appropriate community involvement.
