# Supplementary material for: Koori Quit Pack: A Feasibility Study of a Multi-Component Mailout Smoking Cessation Support for Aboriginal and Torres Strait Islander People: “I Would Recommend it to Anybody. It’s Just so Much Easier.”
Source: Nicotine Tob Res. 2024 May 3;27(3):418–28. doi: 10.1093/ntr/ntae106 (PMC11847770; doi:10.1093/ntr/ntae106)
Supplement: ntae106_suppl_Supplementary_Material [file ntae106_suppl_supplementary_material.docx]

**Supplementary table 1: Cross tabulation and chi-squared test results**

| Variables | Continuous Quitting at 2-weeks | | | Continuous Quitting at 6-months | | | 7-days PPA-Quitting 6 months | | |
| --- | --- | --- | --- | --- | --- | --- | --- | --- | --- |
|  | **No** | **Yes** | **p-value** | **No** | **Yes** | **p-value** | **No** | **Yes** | **p-value** |
| Depressive symptoms at baseline |  |  | 0.856 |  |  | 0.067 |  |  | 0.021* |
| No | 51(79.7%) | 36(78.3%) |  | 56(76.7%) | 19(95%) |  | 45(73.8%) | 30(93.8%) |  |
| Yes | 13(20.3%) | 10(21.7%) |  | 17(23.3%) | 1(5%) |  | 16(26.2%) | 2(6.3%) |  |
| Remoteness |  |  | 0.661 |  |  | 0.915 |  |  | 0.059 |
| Metropolitan area | 34(52.3%) | 26(56.5%) |  | 38(51.4%) | 10(50%) |  | 36(58.1%) | 12(37.5%) |  |
| Remote and rural areas | 31(47.7%) | 20(43.5%) |  | 36(48.6%) | 10(50%) |  | 26(41.9%) | 20(62.5%) |  |
| Socioeconomic disadvantage (SEIFA) |  |  | 0.163 |  |  | 0.167 |  |  | 0.100 |
| Greater socioeconomic disadvantage | 37(56.9%) | 20(43.5%) |  | 46(62.2%) | 9(45%) |  | 40(64.5%) | 15(46.9%) |  |
| No socioeconomic disadvantage | 28(43.1%) | 26(56.5%) |  | 28(37.8%) | 11(55%) |  | 22(35.5%) | 17(53.1%) |  |
| Level of nicotine addiction at baseline |  |  | 0.730 |  |  | 0.663 |  |  | 0.992 |
| Low addiction | 13(20%) | 7(15.2%) |  | 12(16.2%) | 5(25%) |  | 11(17.7%) | 6(18.8%) |  |
| Moderate addiction | 35(53.8%) | 28(60.9%) |  | 45(60.8%) | 11(55%) |  | 37(59.7%) | 19(59.4%) |  |
| High addiction | 17(26.2%) | 11(23.9%) |  | 17(23%) | 4(20%) |  | 14(22.6%) | 7(21.9%) |  |
| Frequency of urges at baseline |  |  | 0.027* |  |  | 0.426 |  |  | 0.880 |
| Low | 30(46.9%) | 12(26.1%) |  | 29(39.7%) | 6(30%) |  | 23(37.1%) | 12(38.7%) |  |
| High | 34(53.1%) | 34(73.9%) |  | 44(60.3%) | 14(70%) |  | 39(62.9%) | 19(61.3%) |  |
| Strength of urges at baseline |  |  | 0.077 |  |  | 0.296 |  |  | 0.421 |
| Low | 26(40%) | 11(23.9%) |  | 24(32.4%) | 9(45%) |  | 20(32.3%) | 13(40.6%) |  |
| High | 39(60%) | 35(76.1%) |  | 50(67.6%) | 11(55%) |  | 42(67.7%) | 19(59.4%) |  |
| Level of motivation to quit at baseline |  |  | 0.606 |  |  | 0.153 |  |  | 0.048* |
| High | 59(90.8%) | 43(93.5%) |  | 67(90.5%) | 20(100%) |  | 55(88.7%) | 32(100%) |  |
| Low | 6(9.2%) | 3(6.5%) |  | 7(9.5%) | 0(0%) |  | 7(11.3%) | 0(0%) |  |
| Level of confidence in quitting at baseline |  |  | 0.377 |  |  | 0.120 |  |  | 0.456 |
| High | 27(41.5%) | 23(50%) |  | 30(40.5%) | 12(60%) |  | 26(41.9%) | 16(50%) |  |
| Low | 38(58.5%) | 23(50%) |  | 44(59.5%) | 8(40%) |  | 36(58.1%) | 16(50%) |  |
| Combination NRT# |  |  | 0.159 |  |  | 0.201 |  |  | 0.581 |
| Yes | 40(64.5%) | 34(77.3%) |  | 20(74.1%) | 7(53.8%) |  | 17(70.8%) | 10(62.5%) |  |
| No | 22(35.5%) | 10(22.7%) |  | 7(25.9%) | 6(46.2%) |  | 7(29.2%) | 6(37.5%) |  |
| Adherent to NRT during pervious quit attempt© |  |  |  |  |  | 0.284 |  |  | 0.542 |
| Yes | **-** | **-** |  | 13(48.1%) | 8(66.7%) |  | 12(50%) | 9(60%) |  |
| No | **-** | **-** |  | 14(51.9%) | 4(33.3%) |  | 12(50%) | 6(40%) |  |
| Type of support used© |  |  | 0.179 |  |  | 0.314 |  |  | 0.151 |
| NRT only | 20(32.3%) | 9(20.5%) |  | 10(37%) | 7(53.8%) |  | 8(33.3%) | 9(56.3%) |  |
| NRT and behavioural support | 42(67.7%) | 35(79.5%) |  | 17(63%) | 6(46.2%) |  | 16(66.7%) | 7(43.8%) |  |

** p-value ≤ 0.05.*

© *For rate of continuous and* 7*‐*day *point prevalence abstinence (PPA) quitting at 6 months, use of NRT, behavioural support and adherence at 10 weeks were used.*

**Additional 2x2 tables:**

**Adherence VS Quitting**

| Outcomes | | Complete case analysis | | Intention to treat analysis | |
| --- | --- | --- | --- | --- | --- |
|  |  | 6-weeks  (n=76) | 10-weeks  (n=48) | 6-weeks  (46.1%) | 10-weeks  (29.1%) |
| Adherent | Yes | 32 (42.1%) | 26(54.2%) | 32 (19.4%) | 26 (15.8%) |
|  | No | 44(57.9%) | 22(45.8%) | 44 (26.7%) | 22 (13.3%) |

**Depression VS Quitting**

| Outcomes | | Baseline  (n=163) | 2-weeks  (n=110) | 6-weeks  (n=78) | 10-weeks  (n=59) |
| --- | --- | --- | --- | --- | --- |
| Depression | Yes | 30 (18.4%) | 17 (15.5%) | 13 (16.7%) | 11 (18.6%) |
|  | No | 133 (81.6%) | 93 (84.5%) | 65 (83.3%) | 48 (81.4%) |

**Vaping VS Quitting**

| Variables | Continuous Quitting at 2-weeks | | | Continuous Quitting at 6-weeks | | | Continuous Quitting at 10-weeks | | |
| --- | --- | --- | --- | --- | --- | --- | --- | --- | --- |
|  | **No** | **Yes** | **p-value** | **No** | **Yes** | **p-value** | **No** | **Yes** | **p-value** |
| E-cigarette use |  |  | 0.714 |  |  | 0.697 |  |  | 0.091 |
| Yes | 7(53.8%) | 6(46.2%) |  | 7(58.3%) | 5(41.7%) |  | 4(80%) | 1(20%) |  |
| No | 58(59.2%) | 40(40.8%) |  | 35(52.2%) | 32(47.8%) |  | 22(40.7%) | 32(59.3%) |  |

**Vaping VS Quit attempt**

| Variables | Continuous Quitting at 2-weeks | | | Continuous Quitting at 6-weeks | | | Continuous Quitting at 10-weeks | | |
| --- | --- | --- | --- | --- | --- | --- | --- | --- | --- |
|  | **No** | **Yes** | **p-value** | **No** | **Yes** | **p-value** | **No** | **Yes** | **p-value** |
| E-cigarette use |  |  | 0.466 |  |  | 0.850 |  |  | 0.428 |
| Yes | 1(7.7%) | 12(92.3%) |  | 1(8.3%) | 11(91.7%) |  | 0(0.0%) | 5(100%) |  |
| No | 15(15.3%) | 77(78.6%) |  | 8(11.9%) | 58(86.6%) |  | 6(11.1%) | 40(74.1%) |  |

**Vaping vs combination NRT**

| Variables | Combination NRT use at 2 weeks | | | Combination NRT use at 6 weeks | | | Combination NRT use at 10 weeks | | |
| --- | --- | --- | --- | --- | --- | --- | --- | --- | --- |
|  | **No** | **Yes** | **p-value** | **No** | **Yes** | **p-value** | **No** | **Yes** | **p-value** |
| E-cigarette with Nicotine |  |  | 0.214 |  |  | 0.634 |  |  | 0.235 |
| Yes | 2(15.4%) | 11(84.6%) |  | 4(36.4%) | 7(63.6%) |  | 0(0.0%) | 3(100%) |  |
| No | 30(32.3%) | 63(67.7%) |  | 19(29.2%) | 46(70.8%) |  | 15(32.6%) | 31(67.4%) |  |

**Vaping vs Adherence**

| Variables | Adherence at 6 weeks | | | Adherence at 10 weeks | | |
| --- | --- | --- | --- | --- | --- | --- |
|  | **Yes** | **No** | **p-value** | **Yes** | **No** | **p-value** |
| E-cigarette with Nicotine |  |  | 0.808 |  |  | 0.654 |
| Yes | 5(45.5%) | 6(54.5%) |  | 2(66.7%) | 1(33.3%) |  |
| No | 27(41.5%) | 38(58.5%) |  | 24(53.3%) | 21(46.7%) |  |
